# Supplementary material for: Feeding a low-protein diet exacerbates the susceptibility to Citrobacter rodentium and dextran sulfate sodium-induced intestinal injury in mice
Source: PLoS One. 2026 Oct 7;21(10):e0335322. doi: 10.1371/journal.pone.0335322 (PMC13645056; doi:10.1371/journal.pone.0335322)
Supplement: Supplementary file 1 [file pone.0335322.s004.docx]

| **Ingredient** | **18% Protein diet (CPD) (g/Kg)** | **1% Protein Diet (LPD) (g/Kg)** |
| --- | --- | --- |
| Casein | 207.0 | 11.5 |
| DL- Methionine | 2.7 | 0.36 |
| Sucrose | 350.0 | 350.0 |
| Corn Starch | 251.3 | 426.54 |
| Maltodextrin | 50.0 | 50.0 |
| Corn Oil | 52.6 | 54.2 |
| Cellulose | 41.06 | 60.6 |
| Vitamin Mix | 10.0 | 10.0 |
| Ethoxyquin | 0.01 | 0.01 |
| Mineral Mix | 13.37 | 13.37 |
| Calcium Phosphate | 17.36 | 22.32 |
| Calcium Carbonate | 4.6 | 1.1 |
