# Supplementary material for: Feeding a low-protein diet exacerbates the susceptibility to Citrobacter rodentium and dextran sulfate sodium-induced intestinal injury in mice
Source: PLoS One. 2026 Oct 7;21(10):e0335322. doi: 10.1371/journal.pone.0335322 (PMC13645056; doi:10.1371/journal.pone.0335322)
Supplement: Supplementary file 2 [file pone.0335322.s005.docx]

|  | **Clinical evaluation** | **Score** |
| --- | --- | --- |
| Body weight (BW) | 20% BW decrease of beginning BW  20% decrease within 48h or 25% of begin BW | 5  35 |
| Dehydration | Normal  Dehydrated | 0  10 |
| Fur quality | Normal  Ruffled  Rash; pallor; redness; icterus; wound; abscess; ulcer; petechiae; ecchymoses | 0  5  10 |
| Respiration | Normal  Increased or decreased; cough; sneezing; Dyspnea (shortness of breath)  Open mouth breathing; cyanosis | 0  5  15 |
| Stool consistency | Normal consistency; volume  Decreased feces; dry; wet and pasty; watery; discoloration  Feces absent  Liquid feces; blood in feces | 0  2  5  10 |
| Hunched back | Normal  Hunched  Hunched; lethargic and no movement when touching | 0  5  10 |
| Movement | Normal  Slight hypoactivity  Hyperactivity; hypoactivity  Ataxia (lack of voluntary coordination of muscle movements); neurologic signs tremors, head tilt)  Reluctant to move; uses cage for support; difficulty getting food or water; seizures  Down with no or minimal movement or response to human; coma | 0  2  5  10  15  35 |
| Grimace scale | Normal  Score 1  Score 2 | 0  5  10 |

A score of 35 or higher was considered the humane endpoint and mice had to be euthanized
