# Supplementary material for: Feeding a low-protein diet exacerbates the susceptibility to Citrobacter rodentium and dextran sulfate sodium-induced intestinal injury in mice
Source: PLoS One. 2026 Oct 7;21(10):e0335322. doi: 10.1371/journal.pone.0335322 (PMC13645056; doi:10.1371/journal.pone.0335322)
Supplement: Supplementary file 3 [file pone.0335322.s006.docx]

| **Antibody** | **Company** | **Cat #** | **Dilution** | **Species** |
| --- | --- | --- | --- | --- |
| CLD-3 | Abcam | ab52231 | 1:2000 | Rabbit |
| OCCLUDIN | Abcam | ab216327 | 1:1000 | Rabbit |
| Beta Actin | Invitrogen | AM4302 | 1:2500 | Mouse |
| Goat anti-mouse | Invitrogen | 62-6520 | 1:1000 |  |
| Mouse anti-rabbit | Santa Cruz | sc-2357 | 1:1000 |  |
| Ki67 | Abcam | Ab15580 | 1:500 | Rabbit |
| Alexa Fluor 488 goat anti-rabbit | Invitrogen | A11008 | 1:1000 |  |
