# Supplementary material for: Feeding a low-protein diet exacerbates the susceptibility to Citrobacter rodentium and dextran sulfate sodium-induced intestinal injury in mice
Source: PLoS One. 2026 Oct 7;21(10):e0335322. doi: 10.1371/journal.pone.0335322 (PMC13645056; doi:10.1371/journal.pone.0335322)
Supplement: Peer review history

# Peer review: Decision Letter 0

**DOI:** 10.1371/journal.pone.0335322.r001

**Authors:** Maria Leonor Oliveira

18 Dec 2025

Feeding a low-protein diet exacerbates the susceptibility to Citrobacter rodentium and Dextran Sulfate Sodium-induced intestinal injury in mice.

Dear Dr. Thind,

Thank you for submitting your manuscript to PLOS ONE. After careful consideration, we feel that it has merit but does not fully meet PLOS ONE’s publication criteria as it currently stands. Therefore, we invite you to submit a revised version of the manuscript that addresses the points raised during the review process.

==============================

We kindly ask you to address all the reviewers’ comments.

Specifically clarify the low protein diet used and discuss the possible effects of carbohydrates on the outcomes observed.

Revise the statistical analyzes and correct any missing symbol for all the figures. Discuss the differences that do not reach statistical significance.

Provide data on CFU counting in feces of C. rodentium infected mice.

Include raw data for all the experiments to accomplish PLoS One publication policy.

Revise Figure legends.

==============================

Please submit your revised manuscript by Feb 01 2026 11:59PM. If you will need more time than this to complete your revisions, please reply to this message or contact the journal office at plosone@plos.org. When you're ready to submit your revision, log on to https://www.editorialmanager.com/pone/ and select the 'Submissions Needing Revision' folder to locate your manuscript file.

- A letter that responds to each point raised by the academic editor and reviewer(s). You should upload this letter as a separate file labeled 'Response to Reviewers'.
- A marked-up copy of your manuscript that highlights changes made to the original version. You should upload this as a separate file labeled 'Revised Manuscript with Track Changes'.
- An unmarked version of your revised paper without tracked changes. You should upload this as a separate file labeled 'Manuscript'.

If you would like to make changes to your financial disclosure, please include your updated statement in your cover letter. Guidelines for resubmitting your figure files are available below the reviewer comments at the end of this letter.

If applicable, we recommend that you deposit your laboratory protocols in protocols.io to enhance the reproducibility of your results. Protocols.io assigns your protocol its own identifier (DOI) so that it can be cited independently in the future. For instructions see: https://journals.plos.org/plosone/s/submission-guidelines#loc-laboratory-protocols. Additionally, PLOS ONE offers an option for publishing peer-reviewed Lab Protocol articles, which describe protocols hosted on protocols.io. Read more information on sharing protocols at https://plos.org/protocols?utm_medium=editorial-email&utm_source=authorletters&utm_campaign=protocols.

We look forward to receiving your revised manuscript.

Kind regards,

Maria Leonor S Oliveira, PhD

Academic Editor

PLOS One

Journal Requirements:

When submitting your revision, we need you to address these additional requirements.

1. Please ensure that your manuscript meets PLOS ONE's style requirements, including those for file naming. The PLOS ONE style templates can be found at

https://journals.plos.org/plosone/s/file?id=wjVg/PLOSOne_formatting_sample_main_body.pdf and https://journals.plos.org/plosone/s/file?id=ba62/PLOSOne_formatting_sample_title_authors_affiliations.pdf

2. Thank you for stating the following in the Acknowledgments Section of your manuscript:

[The authors thank the Imaging Facility at The Hospital for Sick Children for their technical help and support. We also want to thank Bernald Castro for technical support with histological staining. This work would not have been possible without Joel Tan (Dr. John Brumell lab), and Sharon Leung and Emiliano Miraglia (Dr. Peter Kim lab), who provided the wildtype strains of the bacterial pathogens and helped with the microbiology setup in the lab. This research was funded by the Bill & Melinda Gates Foundation (OPP1185057) and the Canadian Institutes of Health Research (CIHR156307).]

We note that you have provided funding information that is currently declared in your Funding Statement. However, funding information should not appear in the Acknowledgments section or other areas of your manuscript. We will only publish funding information present in the Funding Statement section of the online submission form.

Please remove any funding-related text from the manuscript and let us know how you would like to update your Funding Statement. Currently, your Funding Statement reads as follows:

[R.J.B - Bill & Melinda Gates Foundation (OPP1185057) and the Canadian Institutes of Health Research (CIHR156307)

The funders played no role in the study design, data collection and analysis, decision to publish, or preparation of the manuscript.]

Please include your amended statements within your cover letter; we will change the online submission form on your behalf.

3. Thank you for stating the following in the Competing Interests section:

[Amber Farooqui is now employed by Omega Laboratories Inc, a clinical diagnostic laboratory. The other authors declare no competing interests.].

We note that one or more of the authors are employed by a commercial company: Omega Laboratories Inc.

1. Please provide an amended Funding Statement declaring this commercial affiliation, as well as a statement regarding the Role of Funders in your study. If the funding organization did not play a role in the study design, data collection and analysis, decision to publish, or preparation of the manuscript and only provided financial support in the form of authors' salaries and/or research materials, please review your statements relating to the author contributions, and ensure you have specifically and accurately indicated the role(s) that these authors had in your study. You can update author roles in the Author Contributions section of the online submission form.

Please also include the following statement within your amended Funding Statement.

“The funder provided support in the form of salaries for authors [insert relevant initials], but did not have any additional role in the study design, data collection and analysis, decision to publish, or preparation of the manuscript. The specific roles of these authors are articulated in the ‘author contributions’ section.”

If your commercial affiliation did play a role in your study, please state and explain this role within your updated Funding Statement.

2. Please also provide an updated Competing Interests Statement declaring this commercial affiliation along with any other relevant declarations relating to employment, consultancy, patents, products in development, or marketed products, etc.

Within your Competing Interests Statement, please confirm that this commercial affiliation does not alter your adherence to all PLOS ONE policies on sharing data and materials by including the following statement: "This does not alter our adherence to PLOS ONE policies on sharing data and materials.” (as detailed online in our guide for authors http://journals.plos.org/plosone/s/competing-interests).  If this adherence statement is not accurate and there are restrictions on sharing of data and/or materials, please state these. Please note that we cannot proceed with consideration of your article until this information has been declared.

Please respond by return email with an updated Funding Statement and Competing Interests Statement and we will change the online submission form on your behalf.

4. PLOS ONE now requires that authors provide the original uncropped and unadjusted images underlying all blot or gel results reported in a submission’s figures or Supporting Information files. This policy and the journal’s other requirements for blot/gel reporting and figure preparation are described in detail at https://journals.plos.org/plosone/s/figures#loc-blot-and-gel-reporting-requirements and https://journals.plos.org/plosone/s/figures#loc-preparing-figures-from-image-files. When you submit your revised manuscript, please ensure that your figures adhere fully to these guidelines and provide the original underlying images for all blot or gel data reported in your submission. See the following link for instructions on providing the original image data: https://journals.plos.org/plosone/s/figures#loc-original-images-for-blots-and-gels.

In your cover letter, please note whether your blot/gel image data are in Supporting Information or posted at a public data repository, provide the repository URL if relevant, and provide specific details as to which raw blot/gel images, if any, are not available. Email us at plosone@plos.org if you have any questions.

5. Please include captions for your Supporting Information files at the end of your manuscript, and update any in-text citations to match accordingly. Please see our Supporting Information guidelines for more information: http://journals.plos.org/plosone/s/supporting-information.

If the reviewer comments include a recommendation to cite specific previously published works, please review and evaluate these publications to determine whether they are relevant and should be cited. There is no requirement to cite these works unless the editor has indicated otherwise.

[Note: HTML markup is below. Please do not edit.]

Reviewers' comments:

Reviewer's Responses to Questions

**Comments to the Author**

1. Is the manuscript technically sound, and do the data support the conclusions?

Reviewer #1: Yes

Reviewer #2: No

**********

2. Has the statistical analysis been performed appropriately and rigorously?

Reviewer #1: Yes

Reviewer #2: No

**********

3. Have the authors made all data underlying the findings in their manuscript fully available?

The PLOS Data policy

Reviewer #1: No

Reviewer #2: Yes

**********

4. Is the manuscript presented in an intelligible fashion and written in standard English?

Reviewer #1: Yes

Reviewer #2: Yes

**********

Reviewer #1: In this study, authors report preclinical mouse models to show the impact of malnutrition in protection against intestinal dysfunction that can be caused by enteropathogenic bacteria. They used DSS treatment and C. rodentium infection for creating colonic dysfunction in mice and observed that low-protein diet makes animals more susceptible for colonic dysfunction compared to control-protein diet with sufficient protein levels.

Authors support their model by showing that low-protein diet worsens the effects of DSS treatment and increases the infection severity of C. rodentium. They demonstrated that low-protein diet leads to increase in IL-22 expression when infected with C. rodentium and hinder crypt hyperplasia. They also showed that low-protein diet led to decrease in CLD3 expression, a tight junction protein, upon infection by C. rodentium and bacterial translocation of C. rodentium in spleen and liver was much higher in low-protein diet fed mice which they concluded it is caused by loss of colonic barrier integrity.

There are minor issues in the figures that need to be addressed. In Figure S1, panel b presents percent bodyweight change and panel c presents the Clinical Severity Scoring but, it is stated as “(b) Clinical Severity Scoring (CSS), (c) Percent bodyweight change from day 0 and (d) survival was assessed” in the legend. Additionally, there seems to be a formatting problem in Figure 1 and Figure 2 where statistical significance indicators are not visible. I am not sure if the problem occurred during the submission process or during exporting the individual figure files but please make sure the figures are completely accurate.

From my understanding, PLOS journals require all the data including the values used to build the graphs to be made publicly available in the manuscript, the authors provided uncropped Western Blot images from Figure 5 in the supplementary document, but I could not find the individual data values of figures so they need to be added to the manuscript to comply with the data availability policy of PLOS journals.

The manuscript is clearly organized and written and the experimental procedures explained well enough to be reproducible. The conclusions are straightforward with sufficient experimental data to support the findings. Introduction and discussion sections of the manuscript are sufficient to provide an insight to readers with proper acknowledgment of the previous literature.

Reviewer #2: In this manuscript, Thind et al., study the effect of a low protein diet (LPD) on mouse susceptibility to intestinal inflammation caused by Dextran Sulfate Sodium (DSS) and Citrobacter rodentium infection. Authors proposed that LPD exacerbates both DSS- and C. rodentium-induced enteric injury in comparison to a control protein diet (CPD), although the specific mechanisms remain to be fully determined. The paper presents initial evidence that might be of interest in the field. However, there are several major aspects in the study that need to be addressed or clarified before it can be considered for publication.

Major comments:

The authors claim that the observed phenotypes are due to the low protein content in the LPD compared with the CPD. However, the LPD diet contains increased carbohydrate content (corn starch) to compensate for the reduction in protein. Therefore, authors should conduct experiments with additional diet(s) to discard whether the observed phenotypes are protein- or carbohydrate-dependent.

Several conclusions are based on phenotypes or comparisons that do not reach statistical significance (some examples are shown below in the specific points). Authors should include additional biological samples to achieve the statistical significance required for their conclusions.

Specific points:

Fig. 1 and 2. Most signs for statistical significance are missing among panels.

Fig. 2b. Total bacterial counts, obtained by serial dilution plating of fecal samples, should be determined to validate the C. rodentium quantifications by qPCR. This assay is viable considering the strain used is antibiotic (Strep) resistant and this method was used to determine CFUs in liver and spleen (Fig. 5d,e). Also, authors should consider including at least an extra time point (for example, day 10) to determine whether the difference in bacterial colonization is consistent at later time points.

Fig. 2c. How do authors explain the lack of an increase in IL-22 in the CPD control + Citrobacter? Due to the inflammation caused by the pathogen, this proinflammatory cytokine is expected to be augmented independently of the diet used. This is even more relevant considering Fig. 3 shows increased crypt length in the CPD + C. rod group (a sign of inflammation that does not correlate with the low IL-22 levels of Fig. 2c). Authors should consider testing other pro-inflammatory cytokines or inflammation markers to contrast/compare these unexpected observations for IL-22.

Fig. 2e and 3. How do the authors explain a potential increased mortality in the LPD + C. rod group (Figure 2e) if less inflammation (reduced crypt length, Fig. 3a,b) and injury (pathological score, Fig. 3c) are observed in this group compared with CPD + C. rod.

Fig. 4. Although statistically different crypt lengths were observed between the LPD + C. rod and CPD + C. rod (Fig. 3b), no statistical difference among these groups was observed for Ki67+ (Fig. 4b). How do authors explain distinct crypt elongation among these groups in the absence of differences for this cell proliferation marker?

Fig. 5. Graphs in panels b) and c) do not entirely reflect the western blot results shown in a). For example, OCCL in the LPD + C. rod samples is clearly increased compared with the other groups in the western blot but not in the quantification graphs. If this is due to differences in the control protein (B-actin), another experiment with a more homogeneous loading control should be included.

L. 143. “We next determined whether the higher bacterial load and lower survival of LPD mice was associated with…”. The lower survival referred here (Fig. 2e) did not reach statistical significance compared with the other groups (p= 0.07).

L.169 “We found that homogenates of spleen and liver from LPD-fed mice had higher bacterial counts of C. rodentium, consistent with increased translocation”. Only significant for liver, but not spleen.

Minor points:

L149. “Significant crypt hyperplasia as observed by increased crypt length... This typical response to C. rodentium is thought to prevent systemic pathogen spread, and promote repair of the epithelium”. It should be stated in the text that this is also a common hallmark of intestinal inflammation.

Fig. S2c. “Ratio of colon over tail length”. Can authors use a more standard comparison such as colon weight vs length to assess this?

**********

what does this mean?). If published, this will include your full peer review and any attached files.

If you choose “no”, your identity will remain anonymous but your review may still be made public.

**Do you want your identity to be public for this peer review?**  For information about this choice, including consent withdrawal, please see our Privacy Policy

Reviewer #1: No

Reviewer #2: No

**********

[NOTE: If reviewer comments were submitted as an attachment file, they will be attached to this email and accessible via the submission site. Please log into your account, locate the manuscript record, and check for the action link "View Attachments". If this link does not appear, there are no attachment files.]

To ensure your figures meet our technical requirements, please review our figure guidelines: https://journals.plos.org/plosone/s/figures

You may also use PLOS’s free figure tool, NAAS, to help you prepare publication quality figures: https://journals.plos.org/plosone/s/figures#loc-tools-for-figure-preparation.

NAAS will assess whether your figures meet our technical requirements by comparing each figure against our figure specifications.

# Author comment: Author response to Decision Letter 1

**DOI:** 10.1371/journal.pone.0335322.r002

13 Jul 2026

Response to editorial and reviewers’ comments

Editorial comments:

When submitting your revision, we need you to address these additional requirements.

1. Please ensure that your manuscript meets PLOS ONE's style requirements, including those for file naming. The PLOS ONE style templates can be found at

https://journals.plos.org/plosone/s/file?id=wjVg/PLOSOne_formatting_sample_main_body.pdf and https://journals.plos.org/plosone/s/file?id=ba62/PLOSOne_formatting_sample_title_authors_affiliations.pdf

- The main authors went through the requirements and made requested changes where appropriate.

2. Thank you for stating the following in the Acknowledgments Section of your manuscript:

[The authors thank the Imaging Facility at The Hospital for Sick Children for their technical help and support. We also want to thank Bernald Castro for technical support with histological staining. This work would not have been possible without Joel Tan (Dr. John Brumell lab), and Sharon Leung and Emiliano Miraglia (Dr. Peter Kim lab), who provided the wildtype strains of the bacterial pathogens and helped with the microbiology setup in the lab. This research was funded by the Bill & Melinda Gates Foundation (OPP1185057) and the Canadian Institutes of Health Research (CIHR156307).]

We note that you have provided funding information that is currently declared in your Funding Statement. However, funding information should not appear in the Acknowledgments section or other areas of your manuscript. We will only publish funding information present in the Funding Statement section of the online submission form.

Please remove any funding-related text from the manuscript and let us know how you would like to update your Funding Statement.

Please include your amended statements within your cover letter; we will change the online submission form on your behalf.

- The funding information has been removed from the acknowledgements section and added to the cover letter.

- We have updated the funding statement. It now reads as follows:

This work was supported through a grant from the Bill & Melinda Gates Foundation (OPP1185057) and the Canadian Institutes of Health Research (CIHR156307). The funder provided support in the form of salaries for authors [MT, SL, CB], but did not have any additional role in the study design, data collection and analysis, decision to publish, or preparation of the manuscript. The specific roles of these authors are articulated in the ‘author contributions’ section.

3. Thank you for stating the following in the Competing Interests section:

[Amber Farooqui is now employed by Omega Laboratories Inc, a clinical diagnostic laboratory. The other authors declare no competing interests.].

We note that one or more of the authors are employed by a commercial company: Omega Laboratories Inc.

1. Please provide an amended Funding Statement declaring this commercial affiliation, as well as a statement regarding the Role of Funders in your study. If the funding organization did not play a role in the study design, data collection and analysis, decision to publish, or preparation of the manuscript and only provided financial support in the form of authors' salaries and/or research materials, please review your statements relating to the author contributions, and ensure you have specifically and accurately indicated the role(s) that these authors had in your study. You can update author roles in the Author Contributions section of the online submission form.2

Please also include the following statement within your amended Funding Statement.

“The funder provided support in the form of salaries for authors [insert relevant initials], but did not have any additional role in the study design, data collection and analysis, decision to publish, or preparation of the manuscript. The specific roles of these authors are articulated in the ‘author contributions’ section.”

If your commercial affiliation did play a role in your study, please state and explain this role within your updated Funding Statement.

2. Please also provide an updated Competing Interests Statement declaring this commercial affiliation along with any other relevant declarations relating to employment, consultancy, patents, products in development, or marketed products, etc.

Within your Competing Interests Statement, please confirm that this commercial affiliation does not alter your adherence to all PLOS ONE policies on sharing data and materials by including the following statement: "This does not alter our adherence to PLOS ONE policies on sharing data and materials.” (as detailed online in our guide for authors http://journals.plos.org/plosone/s/competing-interests). If this adherence statement is not accurate and there are restrictions on sharing of data and/or materials, please state these. Please note that we cannot proceed with consideration of your article until this information has been declared.

Please respond by return email with an updated Funding Statement and Competing Interests Statement and we will change the online submission form on your behalf.

- For the funding statement, see above.

- Competing interest statement: Amber Farooqui is currently employed by Omega Laboratories Inc, a clinical diagnostic laboratory. This does not alter our adherence to PLOS ONE policies on sharing data and materials. The other authors declare no competing interests.

4. PLOS ONE now requires that authors provide the original uncropped and unadjusted images underlying all blot or gel results reported in a submission’s figures or Supporting Information files. This policy and the journal’s other requirements for blot/gel reporting and figure preparation are described in detail at https://journals.plos.org/plosone/s/figures#loc-blot-and-gel-reporting-requirements and https://journals.plos.org/plosone/s/figures#loc-preparing-figures-from-image-files. When you submit your revised manuscript, please ensure that your figures adhere fully to these guidelines and provide the original underlying images for all blot or gel data reported in your submission. See the following link for instructions on providing the original image data: https://journals.plos.org/plosone/s/figures#loc-original-images-for-blots-and-gels.

In your cover letter, please note whether your blot/gel image data are in Supporting Information or posted at a public data repository, provide the repository URL if relevant, and provide specific details as to which raw blot/gel images, if any, are not available. Email us at plosone@plos.org if you have any questions.

- We added the exact uncropped blot images as supplementary figure 3 and mentioned this in the cover letter.

5. Please include captions for your Supporting Information files at the end of your manuscript, and update any in-text citations to match accordingly. Please see our Supporting Information guidelines for more information: http://journals.plos.org/plosone/s/supporting-information.

If the reviewer comments include a recommendation to cite specific previously published works, please review and evaluate these publications to determine whether they are relevant and should be cited. There is no requirement to cite these works unless the editor has indicated otherwise.

- This has been modified accordingly and all the supplementary captions are provided in the main manuscript.

-Individual supplementary figures and tables are uploaded.

Reviewers’ Comments to the Author

Reviewer #1:

There are minor issues in the figures that need to be addressed. In Figure S1, panel b presents percent bodyweight change and panel c presents the Clinical Severity Scoring but, it is stated as “(b) Clinical Severity Scoring (CSS), (c) Percent bodyweight change from day 0 and (d) survival was assessed” in the legend. Additionally, there seems to be a formatting problem in Figure 1 and Figure 2 where statistical significance indicators are not visible. I am not sure if the problem occurred during the submission process or during exporting the individual figure files but please make sure the figures are completely accurate.

- The figure legend for S1 has been corrected, as well as it is now ensured that all the figures contain the appropriate statistical significance indicators which were missing during the submission process in the last version of the manuscript

- All figures are uploaded individually in the new submission

From my understanding, PLOS journals require all the data including the values used to build the graphs to be made publicly available in the manuscript, the authors provided uncropped Western Blot images from Figure 5 in the supplementary document, but I could not find the individual data values of figures so they need to be added to the manuscript to comply with the data availability policy of PLOS journals.

- We have now uploaded the raw data values used to generate the figures as part of supplementary information

Reviewer #2:

Major comments:

The authors claim that the observed phenotypes are due to the low protein content in the LPD compared with the CPD. However, the LPD diet contains increased carbohydrate content (corn starch) to compensate for the reduction in protein. Therefore, authors should conduct experiments with additional diet(s) to discard whether the observed phenotypes are protein- or carbohydrate-dependent.

This is a valid point. This animal model has limitations, including that changing the relative content of one macronutrient will affect the relative content of at least one of the other macronutrients. We did maintain the same relative content of sugar, i.e., sucrose. We aimed to mimic the carbohydrate-rich and protein-poor staple foods, such as cereals and starches, suboptimal in essential amino acids, that malnourished children in low-income countries are generally exposed to. It is a model we describe in previously published work (Hu et al., Nat Comm 2022) and resembles a model developed by Brown et al., Nat Comm 2015. This study did not aim to determine if there is a specific contribution of a difference in starch on our phenotype and those experiments fall outside of the scope of the current manuscript. We added the limitations of the model to the discussion on page 18 line 448-452.

Several conclusions are based on phenotypes or comparisons that do not reach statistical significance (some examples are shown below in the specific points). Authors should include additional biological samples to achieve the statistical significance required for their conclusions.

Specific points:

Fig. 1 and 2. Most signs for statistical significance are missing among panels.

- it is now ensured that all the figures contain the appropriate statistical significance indicators which did not transfer during the submission process in the last version of the manuscript

- All figures are uploaded individually in the new submission with the appropriate statistical significance indicators

Fig. 2b. Total bacterial counts, obtained by serial dilution plating of fecal samples, should be determined to validate the C. rodentium quantifications by qPCR. This assay is viable considering the strain used is antibiotic (Strep) resistant and this method was used to determine CFUs in liver and spleen (Fig. 5d,e). Also, authors should consider including at least an extra time point (for example, day 10) to determine whether the difference in bacterial colonization is consistent at later time points.

We thank the reviewer for this suggestion. To validate the qPCR-based quantification of C. rod in fecal samples, we have added the serial dilution plating data for day 7 stools. As shown in Fig. S2d, the CFU-based measurement closely correlates with the qPCR results. For the liver and spleen, the qPCR method has not been validated, hence we were limited to the traditional culturing method for this.

At this moment we are unable to look at the response day 10 post-infection (highlighted in the limitation section – line 444-446). In this regard, weanling mice in this study are infected on day 7 of the experimental period, and sacrificed 7 days later. This is based on their weight loss trajectories affecting the duration of the experimental period due to ethical constraints.

Fig. 2c. How do authors explain the lack of an increase in IL-22 in the CPD control + Citrobacter? Due to the inflammation caused by the pathogen, this proinflammatory cytokine is expected to be augmented independently of the diet used. This is even more relevant considering Fig. 3 shows increased crypt length in the CPD + C. rod group (a sign of inflammation that does not correlate with the low IL-22 levels of Fig. 2c). Authors should consider testing other pro-inflammatory cytokines or inflammation markers to contrast/compare these unexpected observations for IL-22.

It is plausible that the IL-22 response is earlier in the CPD mice as compared to the LPD mice. This may explain the crypt hyperplasia response by day 7 post infection in the CPD mice as compared to the LPD rather than the high IL-22 at this timepoint. IL-22 response is acute and later triggers the epithelial repair programs to prevent systemic bacterial translocation. This includes increased number of Ki67+ intestinal epithelial cells and crypt height to promote barrier repair and a return to homeostasis, at which point its levels drop (Keir et al., 2020). This reflects exactly what we see in the CPD-fed mice. In addition, crypt hyperplasia can also occur independently of the IL-22 response such as through IFN-γ response (Stamnaes et al., 2025), which was beyond the scope of this study to look at.

The comment on looking at additional pro-inflammatory cytokines/inflammation markers is a valid point. Line 468-470 reads, “Other elements of the inflammatory, metabolic and organ response, including cytokine profiles, serum albumin levels, blood glucose, and liver and kidney functions were also not examined, but have been shown to be altered in clinical and preclinical models of SM” to reflect this comment. The primary objective of this study was “to describe novel mouse models of severe wasting and large intestinal infection and inflammation and assess the impact on intestinal epithelial outcomes”, with IL-22 selected based on its well-established role in epithelial barrier function and host defense during enteric infection.

Fig. 2e and 3. How do the authors explain a potential increased mortality in the LPD + C. rod group (Figure 2e) if less inflammation (reduced crypt length, Fig. 3a,b) and injury (pathological score, Fig. 3c) are observed in this group compared with CPD + C. rod.

While intestinal inflammation and injury are key determinants of disease severity during C. rodentium infection, mortality is not solely influenced by the magnitude of local colonic pathology. Crypt hyperplasia is an essential response in the context of an intestinal infectious or inflammatory insult, and lack of a crypt hyperplastic response has been shown to impair barrier function and allow for increased bacterial translocation￼￼. This could be one of the mechanisms behind the increased vulnerability in our LPD-fed mice. In addition, as we have published previously(Thind et al., 2024)￼, LPD-fed mice have an impaired capacity to mount effective systemic immune responses (hypo-responsiveness) which may lead to a less infective response to a C Rod infection in our model and thereby increased mortality following c. rodentium infection. This is discussed in detail in the discussion section.

Fig. 4. Although statistically different crypt lengths were observed between the LPD + C. rod and CPD + C. rod (Fig. 3b), no statistical difference among these groups was observed for Ki67+ (Fig. 4b). How do authors explain distinct crypt elongation among these groups in the absence of differences for this cell proliferation marker?

We thank the reviewer for this valuable comment. It is important to note that crypt hyperplasia is a reflection of a multitude of epithelial processes, including but not limited to, epithelial proliferation (as measured by Ki67 in this study), and cellular differentiation and turnover(Duckworth & Pritchard, 2009; Huan et al., 2017) as stated in the discussion section. However, examining these mechanisms was beyon

Attachment Submitted filename: plosone crod reviewer comments v2.docx

# Peer review: Decision Letter 1

**DOI:** 10.1371/journal.pone.0335322.r003

**Authors:** Maria Leonor Oliveira

17 Aug 2026

Feeding a low-protein diet exacerbates the susceptibility to Citrobacter rodentium and Dextran Sulfate Sodium-induced intestinal injury in mice.

PONE-D-25-53818R1

Dear Dr. Thind,

We’re pleased to inform you that your manuscript has been judged scientifically suitable for publication and will be formally accepted for publication once it meets all outstanding technical requirements.

Within one week, you’ll receive an e-mail detailing the required amendments. When these have been addressed, you’ll receive a formal acceptance letter and your manuscript will be scheduled for publication.

An invoice will be generated when your article is formally accepted. Please note, if your institution has a publishing partnership with PLOS and your article meets the relevant criteria, all or part of your publication costs will be covered. Please make sure your user information is up-to-date by logging into Editorial Manager at Editorial Manager® and clicking the ‘Update My Information' link at the top of the page. For questions related to billing, please contact billing support.

If your institution or institutions have a press office, please notify them about your upcoming paper to help maximize its impact. If they’ll be preparing press materials, please inform our press team as soon as possible -- no later than 48 hours after receiving the formal acceptance. Your manuscript will remain under strict press embargo until 2 pm Eastern Time on the date of publication. For more information, please contact onepress@plos.org.

Kind regards,

Maria Leonor S Oliveira, PhD

Academic Editor

PLOS One

Additional Editor Comments (optional):

Reviewers' comments:

Reviewer's Responses to Questions

**Comments to the Author**

Reviewer #1: All comments have been addressed

Reviewer #2: All comments have been addressed

**********

2. Is the manuscript technically sound, and do the data support the conclusions?

Reviewer #1: Yes

Reviewer #2: Partly

**********

3. Has the statistical analysis been performed appropriately and rigorously?

Reviewer #1: Yes

Reviewer #2: Yes

**********

4. Have the authors made all data underlying the findings in their manuscript fully available?

The PLOS Data policy

Reviewer #1: Yes

Reviewer #2: Yes

**********

5. Is the manuscript presented in an intelligible fashion and written in standard English?

Reviewer #1: Yes

Reviewer #2: Yes

**********

Reviewer #1: The study has some limitations especially with the in vivo infection models and the authors tried to address the limitations in the manuscript as best as they can. Their approach is adjusting the carbohydrate levels in the diet while altering the protein levels which can have a big impact on the infection severity independent of the protein levels in the diet. They also focus on specific markers of inflammation rather than having a multi-layered approach.

Overall, authors answered the concerns of reviewers and made some modifications accordingly. All the limitations and the preferences of authors considered, the manuscript is sufficient to support their conclusion and their infection model.

Reviewer #2: The authors have answered all my questions and adequately addressed my points.

I have no additional comments.

**********

what does this mean?). If published, this will include your full peer review and any attached files.

If you choose “no”, your identity will remain anonymous but your review may still be made public.

**Do you want your identity to be public for this peer review?**  For information about this choice, including consent withdrawal, please see our Privacy Policy

Reviewer #1: No

Reviewer #2: No

**********

# Editor report: Acceptance letter

**DOI:** 10.1371/journal.pone.0335322.r004

**Authors:** Maria Leonor Oliveira

PONE-D-25-53818R1

PLOS One

Dear Dr. Thind,

I'm pleased to inform you that your manuscript has been deemed suitable for publication in PLOS One. Congratulations! Your manuscript is now being handed over to our production team.

At this stage, our production department will prepare your paper for publication. This includes ensuring the following:

* All references, tables, and figures are properly cited

* All relevant supporting information is included in the manuscript submission,

* There are no issues that prevent the paper from being properly typeset

You will receive further instructions from the production team, including instructions on how to review your proof when it is ready. Please keep in mind that we are working through a large volume of accepted articles, so please give us a few days to review your paper and let you know the next and final steps.

Lastly, if your institution or institutions have a press office, please let them know about your upcoming paper now to help maximize its impact. If they'll be preparing press materials, please inform our press team within the next 48 hours. Your manuscript will remain under strict press embargo until 2 pm Eastern Time on the date of publication. For more information, please contact onepress@plos.org.

You will receive an invoice from PLOS for your publication fee after your manuscript has reached the completed accept phase. If you receive an email requesting payment before acceptance or for any other service, this may be a phishing scheme. Learn how to identify phishing emails and protect your accounts at https://explore.plos.org/phishing.

If we can help with anything else, please email us at customercare@plos.org.

Thank you for submitting your work to PLOS One and supporting open access.

Kind regards,

PLOS One Editorial Office Staff

on behalf of

Dr. Maria Leonor S Oliveira

Academic Editor

PLOS One
